# Role of Digoxin-Like Immunoreactive Substance in the Pathogenesis of Transient Tachypnea of Newborn

**DOI:** 10.1155/2013/704763

**Published:** 2013-07-07

**Authors:** Mehmet Yalaz, Erturk Levent, Murat Olukman, Sebnem Calkavur, Mete Akisu, Nilgun Kultursay

**Affiliations:** ^1^Division of Neonatology, Department of Pediatrics, Faculty of Medicine, Ege University, Bornova, Izmir, Turkey; ^2^Division of Pediatric Cardiology, Department of Pediatrics, Faculty of Medicine, Ege University, Turkey; ^3^Department of Pharmacology, Faculty of Medicine, Ege University, Turkey

## Abstract

*Background.* Transient tachypnea of newborn (TTN) is usually observed in term or near-term infants. It constitutes an important part of the respiratory distress cases observed in the neonatal intensive care unit (NICU). *Aim.* This paper examines the effects of digoxin-like immunoreactive substance (DLIS) on fluid and ion balance, hemodynamic and echocardiographic parameters of neonates with TTN. *Methods.* Plasma DLIS, Na^+^, K^+^, urea, creatinine, serum and urine osmolarity, urine FeNa^+^, 24-hour urine output, echocardiographic investigation and mean blood pressure, and clinical parameters of disease severity were recorded in TTN group and compared with control on the 1st and 7th days of their lives. *Results.* Plasma DLIS levels were statistically higher in TTN group (0.66 ± 0.37 ng/mL) compared to control group (0.24 ± 0.20 ng/mL) both on the 1st day (*P* < 0.01) and the 7th day (*P* < 0.05). For TTN group, significant correlation was found between plasma DLIS levels and maximum respiratory rate, duration of tachypnea, and length of hospitalization on the 1st day. Plasma DLIS levels were correlated negatively with serum osmolarity levels. Plasma DLIS levels were positively correlated with urine output, urinary FeNa^+^ levels, cardiac output, left ventricles end diastolic diameters, and right ventricles end diastolic diameters. *Conclusions.* Increased DLIS levels were correlated with disease severity in cases with TTN. This increase may be a primary or secondary event in the disease progress. It may help reduce the fluid overload due to already disturbed cardiac functions in patients by increasing urine output and natriuresis; however it may also contribute to disease pathogenesis, by inhibiting alveolar Na^+^-K^+^-ATPase which further decreases fetal alveolar fluid resorption.

## 1. Introduction

Although transient tachypnea of newborn (TTN) is usually observed in term or near-term infants, it constitutes an important part of the respiratory distress cases observed in the neonatal intensive care unit (NICU) accounting for 5–30% of all NICU admissions [1–3]. Usually, it is characterized by a clinically benign tachypnea and oxygen requirement self-limiting within a few days; however, in some cases it may exert a more serious clinical course [3]. The underlying mechanism of TTN is thought to be a delay in the resorption of fetal lung fluid, and cesarean section increases the risk of TTN. However, the exact pathogenesis is still not clear [3–7]. 

Some recent studies suggested that a defect in the pulmonary epithelial sodium transport, occurring due to the dysfunction of some ion channels on alveolar epithelial cells and causing a delay in resorption of fetal lung fluid, may be responsible for the pathogenesis of TTN [8, 9]. Amiloride-sensitive epithelial sodium channels (ENaC) and sodium-potassium adenosine triphosphates (Na^+^-K^+^-ATPase) play an essential role in the pulmonary sodium transport [8, 10].

Na^+^ absorption by ENaC is activated at the time of birth, Na^+^ moves into the interstitium via basolateral Na^+^-K^+^-ATPase, and water flows passively along this osmotic gradient through paracellular and intracellular spaces [8, 9, 11]. The process is accelerated at the onset of labor, along with hormonal effects, such as thyroid hormones, glucocorticoids, and catecholamines. Beta adrenergic receptor (ADRB) encoding genes have been shown to predispose TTN [12]. 

Water channel aquaporin 5 (AQP5) facilitates the majority of water transport across the apical membrane of alveolar epithelia. AQP5 expression has been observed to be higher in tracheal aspirates of TTN cases, possibly as a compensatory mechanism, while *β*-ENaC expression was found to be lower in neonates with Respiratory Distress Syndrome (RDS) [13]. N-terminal pro-B-type natriuretic peptide (NT-proBNP) levels were found to be higher in cases when TTN is related to the disease severity [14].

 Cardiac influences of TTN, such as ventricular dysfunction, increased pulmonary vascular, and central venous pressure, are described [1, 14]. 

 Endogen glycosides (digoxin-like immunoreactive substance (DLIS)), first described by Gruber et al. [15], are adrenocortical hormones, which are the biologic active inhibitors of Na^+^-K^+^-ATPase [16]. Various clinical and laboratory investigations have shown that DLIS is directly related to the sodium homeostasis, volume overload, natriuresis, and blood pressure [16]. In some disorders, such as hypertension, acromegaly, liver diseases, and renal and cardiac failure, high DLIS level was observed in the blood, despite no digoxin use [17]. In newborns, especially in preterm infants, unrelated to the maternal and placental levels, DLIS levels were reported to be much higher than the other pediatric age groups [18–21].

 The aim of this study is to investigate the cardiac, pulmonary, and hemodynamic effects of DLIS in TTN and if it may have a role in the pathogenesis of disease.

## 2. Materials and Methods

In this prospective study, 15 patients with TTN, who were hospitalized in Ege University Children's Hospital, Neonatal Intensive Care Unit (NICU), between April 2000 and June 2003, were included. Fifteen healthy control infants were also prospectively included in the study for comparison. One gestational age matched infant was taken as a control for every TTN patient. The study was approved by the local Ethics Committee. An informed written consent was obtained from the parents before inclusion of their children in the study.

### 2.1. Inclusion Criteria

Infants born at ≥34-week gestational age, with clinical signs, chest X-ray findings, and clinical course consistent with TTN, were enrolled. Infants with respiratory distress were diagnosed as TTN, if they fulfilled the following criteria [22]: (i) onset of respiratory distress (tachypnea, retractions, grunting, nasal flaring, and mild cyanosis) within six hours after birth; (ii) persistence of respiratory distress beyond 12 hours after birth; and (iii) chest X-ray consistent with TTN (perihilar streaking, hyperinflated lungs, flattening of the diaphragm, and fluid in the fissures).

### 2.2. Exclusion Criteria

The subjects were excluded from the study under the following criteria: digoxin treatment for the infant or mother, persistent hypoglycemia, infants of diabetic mothers, hypoglycemia, polycythemia, meconium aspiration, congenital heart disease, hemodynamically significant patent ductus arteriosus (PDA), major congenital anomalies, perinatal or postnatal asphyxia (5-minute Apgar score < 7, pH < 7.10, and HCO_3_ < 15 mmol), preeclampsia, prolonged rupture of membranes (>18 hours), chorioamnionitis, and other causes of respiratory distress (intrauterine pneumonia, aspiration, respiratory distress syndrome, pneumonia, meconium aspiration, polycythemia, hypoglycemia, and early onset sepsis).

### 2.3. Study Design and Fluid-Electrolyte Balance Parameters

All the study group infants had the same intravenous fluid, total parenteral nutrition (TPN), and follow-up protocols. After obtaining the informed consent from families on the 1st day (day of admission) before the initiation of any medical treatment and on the 7th day, plasma DLIS levels together with hemogram (Cell-Dyn 3700SL, Hemocounter, Germany), blood gas analysis (Phox Plus L Autoanalyser, Nova Biomedical, USA), serum sodium (Na^+^), potassium (K^+^), urea, creatinine, (Synchron CX9 Clinical System Autoanalyser, Beckman Coulter, USA), and blood and urine osmolarity (Freezing-point Technology, The Advanced Micro-Osmometer, USA), were measured. 

During the study days, 24-hour urine was collected, and the quantity and fractioned Na^+^ excretion (FeNa^+^) were calculated [23]. 

Serum C-reactive protein (CRP) (Nephelometry, Dade-Behring, BNII, Germany) and blood culture antibiogram (Bact-T Alert, Biomérieux, France) were also evaluated in order to exclude pneumonia and infection.

The amount of fluid given to the mother before delivery, demographic characteristics (sex, birth weight, gestational age, delivery type, and antenatal steroids), clinical characteristics (Apgar scores at 1st and 5th minutes), respiratory rate on admission (RR_0_), maximum respiratory rate on follow-up (RR_max⁡_), duration of tachypnea (DoT), the need for respiratory assistance, and the duration of hospitalization (DoH) were all recorded.

### 2.4. Sampling and Measurement of Plasma DLIS Levels

One mL venous blood samples were collected in sodium EDTA tubes for plasma DLIS measurements. According to the recommendations of the manufacturing company, sera were stored at −20°C until the study day within the maximum storage duration of six months. DLIS levels were measured with Enzyme Multiplied Immune Technology method (COBAS Mira Ins., Branchburg, NJ, USA) using Hitachi 912 (Japan). In this type of assay, a sample of interest with the analyte is added to a fixed quantity of enzyme-bound drug and the antidrug antibody. After the addition of substrate, absorbance measurements are taken at time intervals to determine the speed of the enzyme reaction. The more the free analyte in the sample, the faster the enzyme reaction because only the unbound enzyme-drug complexes are capable of binding the substrate. The method can be used for whole blood, serum, or urine. Enzyme multiplied immunoassays can be fully automated with a fast throughput of clinical samples especially in laboratories specialized in monitoring therapeutic drugs [24]. This technique prevents the occurrence of any type of cross-reaction with drugs and reactants (steroid structured hormone compound, furosemide, dopamine, aubaine, caffeine, acetyl salicylic acid, etc.) but only with digoxin. Results of DLIS levels are expressed as ng/mL.

### 2.5. Hemodynamic-Echocardiographic Investigations

All patients were echocardiographically evaluated on calm setup and supine position by the same pediatric cardiologist using Hewlett-Packard Sonos 1000 System Echocardiography Device (USA) with 7.5 Mhz transducer. Structural two-dimension evaluation of heart was completed with M-mode, two-dimensional, and Doppler echocardiographic evaluation of hemodynamic functions. All evaluations were video recorded and repeated three times for each investigation on the 1st and 7th days of life, and the mean values were calculated and recorded. Left and right ventricle evaluations were performed on the short axis parasternally, on the mitral valve level with M-mode. For left and right ventricles (LV, RV), end diastolic diameters (LVEDD and RVEDD) and end systolic diameters (LVESD and RVESD) were also measured. Parasternal long axis measurements of aorta and left atrial diameter (LAD), at the aortic valve level during systole period, were estimated (LA/Aorta). Fractional shortening (FS) was calculated by using [(LV_EDD_ − LV_ESD_)/LV_EDD_ × 100] formula, and ejection fraction (EF) was calculated by using [(LV_EDD_^3^ − LV_ESD_^3^)/LV_EDD_^3^ × 100] formula. Cardiac output (CO) was calculated with special program of echocardiography using [(end diastolic volume − end systolic volume)/1000] × heart rate] formula for each infant. Doppler flow values were obtained from mitral wave data observed from apical 2-space. All the measurements were performed in accordance with the *American Society of Echocardiography* guidelines [25] and Silverman's reference study [26].

Mean blood pressure (mBP, mmHg) measurements were made 3 times on the study days with oscillometric technique (ARGUS LCM, Schiller AG, Switzerland) when the infants were in supine position in calm awake state. Heart rate (HR) recorded during echocardiographic study was also used.

### 2.6. Statistical Analysis

All data were analyzed using the SPSS 17.0 Software for Windows (SPSS Int. Co., USA). All values were provided as median, minimum-maximum, and mean ± standard deviation. The statistical evaluations were performed using paired *t*-test, unpaired *t*-test, Pearson and Spearman correlation tests, and Mann Whitney *U*-test. Parameters with *P* < 0.05 were considered statistically significant.

## 3. Results

Out of 1296 patients hospitalized in NICU during the three-year study period, 61 infants (4.7%) were diagnosed with TTN. The study group consisted of 15 eligible patients, who were granted permission for inclusion in the study by their parents. Fifteen healthy infants, born in the same hospital and having similar demographic characteristics, were also evaluated as control group.

### 3.1. Demographic and Perinatal Characteristics

The amount of fluid given to the mother before delivery, demographic characteristics (sex, birth weight, gestational age, delivery types, and antenatal steroids), and clinical characteristics (Apgar scores at 1st and 5th minutes, RR_0_, RR_max⁡_, DoT, the need for respiratory assistance, DoH, and CRP) were all observed to be similar in both groups. None of them had blood culture positivity (Table 1).

The TTN group was found to have higher RR_0_, RR_max⁡_, and DoH than control group (*P* < 0.05). Two patients (13.3%) developed pneumothorax and thus needed mechanical ventilation. Other two infants (13.3%) needed CPAP treatment. None of the patients were on oxygen at day 7 (Table 2).

### 3.2. DLIS Levels

**Figure 1:**
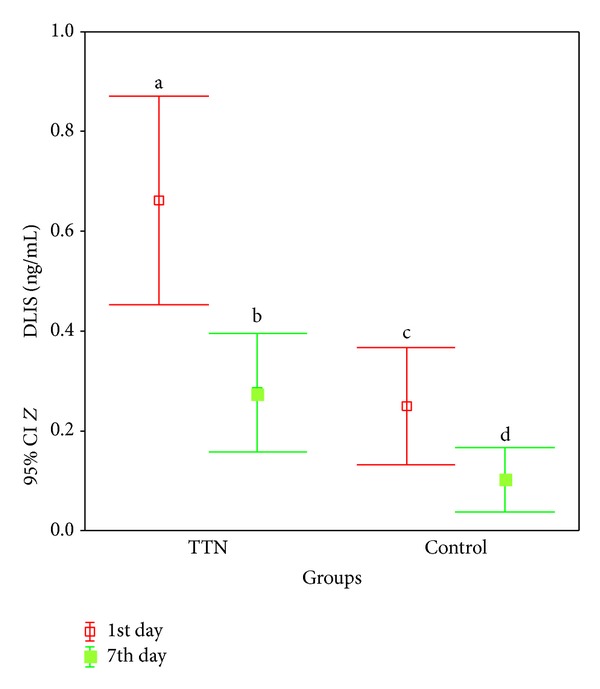
According to the time, DLIS (ng/mL) levels of TTN and control groups for (a versus b) and (a versus c) *P* < 0.01 and for (c versus d) and (b versus d) *P* < 0.05.

On the 1st day, the TTN group had higher DLIS levels (0.66 ± 0.37 ng/mL) compared to the control group (0.24 ± 0.20 ng/mL) (*P* < 0.01). This difference became more prominent on the 7th day of life (0.27 ± 0.21 ng/mL versus 0.11 ± 0.11 ng/mL; *P* < 0.05) (Table 1, Figure 1).

DLIS levels decreased on the 7th day compared to the 1st day of life (*P* < 0.01). This decrease became more prominent in TTN group (*P* < 0.05) (Table 1, Figure 1).

### 3.3. Fluid and Electrolyte Parameters

#### 3.3.1. Comparison of the 1st and 7th Day Values

*TTN Group. *Serum Na^+^ values on the 1st day (143.0 ± 4.1 meq/L) were higher than those on the 7th day (139.7 ± 5.2 meq/L) (*P* < 0.05). Similarly, urine FeNa^+^ values were initially higher than those on the 7th day (2.47 ± 0.26% versus 1.79 ± 0.25%) (*P* < 0.001). Diuresis decreased from 3.12 ± 0.82 mL/kg/h to 2.05 ± 0.41 mL/kg/h (*P* < 0.001), and serum osmolarity reduced from 304.1 ± 6.9 mosm/L to 297.3 ± 7.5 mosm/L (*P* < 0.05). Other parameters were similar on the 1st day and the 7th day (Table 2).

*Control Group.* Serum Na^+^ (139.3 ± 5.1 meq/L) and urine FeNa^+^ (1.76 ± 0.34%) levels decreased to 135.6 ± 4.6 meq/L and 1.51 ± 0.29% (*P* < 0.05; *P* < 0.01), respectively. Diuresis did not change, but blood urea levels decreased (30.4 ± 8.6 mg/dL versus 40.2 ± 9.9 mg/dL) (*P* < 0.05) (Table 2).

#### 3.3.2. Comparison of Groups

*On the 1st Day.* Serum Na^+^ (143.0 ± 4.1 meq/L), FeNa^+^ (2.47 ± 1.76%), diuresis (3.12 ± 0.82 mL/kg/h), and serum osmolarity (304.1 ± 6.9 mosm/L) levels were higher in the TTN group compared to the control group (139.3 ± 5.1 meq/L; 1.76 ± 0.34%; 2.34 ± 0.82 mL/kg/h; 295.8 ±  10.1 mosm/L) (*P* < 0.05; *P* < 0.001; *P* < 0.05; *P* < 0.05), respectively. Only K^+^ values were lower in the TTN group (3.7 ± 0.4 meq/L versus 4.0 ± 0.2 meq/L) (*P* < 0.05) (Table 2).

*On the 7th Day. *The TTN group had higher Na^+^ (139.3 ± 5.1 meq/L), FeNa^+^ (1.79 ± 0.25%), blood urea (40.2 ± 9.9 mg/dL), and urine osmolarity (402.7 ± 40.6 mosm/L) levels than the control group (135.6 ± 4.6 meq/L, 1.51 ± 0.29%, 28.6 ± 7.8 mg/dL, and 364.0 ± 54.9 mosm/L) (*P* < 0.05; *P* < 0.01; *P* < 0.01; *P* < 0.05), respectively (Table 2).

### 3.4. Hemodynamic-Echocardiographic Parameters

#### 3.4.1. Comparison of the 1st and 7th Day Values

*TTN Group. *CO, EF, LVEDD, LVESD, RVEDD, RVESD, LA/aorta ratio, and HR levels were all significantly different on the 1st day compared to the 7th day (*P* < 0.01, *P* < 0.01, *P* < 0.01, *P* < 0.001, *P* < 0.05, *P* < 0.05, *P* < 0.001, and *P* < 0.001), respectively. However, FS and mBP did not change within time (Table 3).

*Control Group. *Only EF was statistically different on the 1st day compared to the 7th day (49.0 ± 2.0% versus 43.0 ± 3.0%) (*P* < 0.05) (Table 3).

#### 3.4.2. Comparison of Groups

*On the 1st Day. *The TTN group had higher CO, EF, LVEDD, LVESD, RVEDD, RVESD, LA/aorta ratio, HR, and mBP values (*P* < 0.05, *P* < 0.05, *P* < 0.05, *P* < 0.001, *P* < 0.01, *P* < 0.05, *P* < 0.001, *P* < 0.001, and *P* < 0.01), respectively (Table 3). 

*On the 7th Day. *TTN group had statistically significantly higher value, of LVESD (12.92 ± 1.23 versus 11.89 ± 0.72 mm/kg) (*P* < 0.01) and RVEDD (23.66 ± 2.72 versus 20.88 ± 1.47 mm/kg) (*P* < 0.01) (Table 3).

### 3.5. Correlation Analysis

#### 3.5.1. DLIS Values in Relation to Time

**Figure 2:**
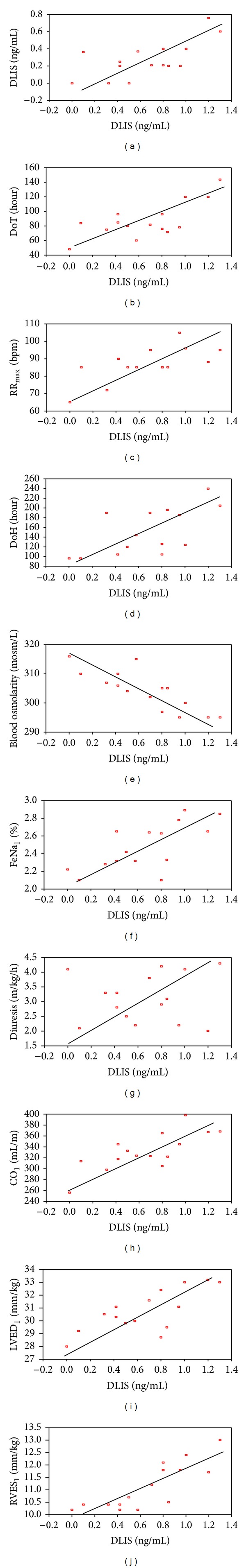
The significant positive correlation was found between DLIS_1_ levels and plasma DLIS_7_ ((a) *r* = 0.605, *P* < 0.01), duration of tachypnea ((b) *r* = 0.713, *P* < 0.01), RR_max⁡_ ((c) *r* = 0.655, *P* < 0.01), duration of hospitalization ((d) *r* = 0.655, *P* < 0.01), FeNa_1_ ((f) *r* = 0.717, *P* < 0.01), diuresis at the 1st day ((g) *r* = 0.755, *P* < 0.01), CO_1_ ((h) *r* = 0.763, *P* < 0.01), LVED_1_ ((i) *r* = 0.740, *P* < 0.01), and RVES_1_ ((j) *r* = 0.828, *P* < 0.001), but significant negative correlation was found between DLIS_1_ levels and blood osmolarity at the 1st day ((e) *r* = −0.837, *P* < 0.001).

DLIS values at the 1st and 7th days were in correlation in theTTN group (*r* = 0.605, *P* < 0.01) (Figure 2(a)) but not in the control group.

#### 3.5.2. DLIS Values in Relation to Clinical Findings

Plasma DLIS values at the 1st day (DLIS_1_) were correlated with DoT (*r* = 0.713, *P* < 0.01), RR_max⁡_ (*r* = 0.655, *P* < 0.01), and DoH (*r* = 0.655, *P* < 0.01) (Figures 2(b), 2(c), and 2(d)). However, DLIS_1_ values were not correlated with the amount of fluid given to the mother before delivery, demographic characteristics (sex, birth weight, gestational age, delivery type, and antenatal steroids), and clinical characteristics (Apgar scores at 1st and 5th minutes, RR_0_, the need for respiratory assistance, and CRP levels). No correlation was observed between plasma DLIS_7_ levels and any of the above mentioned parameters.

#### 3.5.3. DLIS Values in Relation to Fluid and Electrolyte Parameters

TTN group DLIS_1_ levels were found to be in negative correlation with blood osmolarity (*r* = −0.837, *P* < 0.001) but in positive correlation with FeNa^+^ (*r* = 0.717, *P* < 0.01) and diuresis (*r* = 0.755, *P* < 0.01) (Figures 2(e), 2(f), and 2(g)). Plasma DLIS_7_ values were found to be negatively correlated only with blood urea levels (*r* = −0.608, *P* < 0.05). 

For control group, plasma DLIS levels had no correlation with any of the parameters at the 1st and 7th days.

#### 3.5.4. DLIS Values in Relation to Hemodynamic-Echocardiographic Parameters

In TTN group, plasma DLIS_1_ values were positively correlated with CO (*r* = 0.763, *P* < 0.01), LVEDD (*r* = 0.740, *P* < 0.01), and RVESD (*r* = 0.828, *P* < 0.001) (Figures 2(h), 2(i), and 2(j)). Other parameters had no correlation in both groups during any of the study days.

## 4. Discussion

TTN is not always a benign condition. In our study group of 15 cases, about 13.3% required nasal CPAP, another 13.3% required mechanical ventilation, and other 13.3% of cases had pneumothorax. The pathophysiology of TTN is not adequately explained, and a delay in the resorption of fetal alveolar fluid is thought to be a major problem [1, 4, 11, 12, 27]. Recently, some studies attributed the pulmonary epithelial sodium transport defects for this delayed absorption. The role of Na^+^-K^+^-ATPase in alveolar fluid clearing has been shown in animal models [28–30]. Besides, DLIS has been shown as an inhibitor of Na^+^-K^+^-ATPase in in vitro and in vivo studies [15–18, 31, 32]. However, its role in TTN pathophysiology has not been investigated so far.

Among the adult patients of intensive care unit (ICU), higher mean plasma DLIS levels (0.52 ± 0.36 ng/mL) were closely related to increased Acute Physiology and Chronic Health Evaluation (APACHE) II scores (*P* < 0.001) and higher mortality rates (*P* < 0.01) compared to the patients with immeasurably low DLIS levels [16].

DLIS levels were reported to be high and around 0.41–0.56 ng/mL in the healthy term infants at the time of birth, unrelated to birth weight, sex, and gestational age. These high DLIS levels decrease to immeasurable levels within 14–45 days [19, 33–35]. In our control group of healthy term infants, the mean 1st day plasma DLIS levels (0.24 ± 0.20 ng/mL) were higher than the 7th day values (0.11 ± 0.11 ng/mL). However, TTN group had much higher initial DLIS levels (0.66 ± 0.37 ng/mL) compared to control group (0.24 ± 0.20 ng/mL). Even on the 7th day, the values (0.27 ± 0.21 ng/mL) were still higher than the initial values (0.24 ± 0.20 ng/mL) of control group. Gestational age, sex, birth weight, fluid amount given to the mother before delivery, and antenatal steroids were all unrelated to the plasma DLIS levels in both groups. There was a positive correlation between RR_max⁡_, DoT, DoH, and DLIS levels, showing the close relation of DLIS with the severity of TTN. DLIS values were not related to CRP levels; this suggested that it does not act as an acute phase reactant, and it may also be a causative factor in TTN pathophysiology rather than being a consequence.

Na^+^-K^+^-ATPase mRNA levels are the highest in postnatal first three days (during which the fluid resorption is maximum) and stay high up to the 14th day compared to adult levels [36, 37]. Therefore, the high plasma DLIS levels in the first days of life, as observed in our study, may have prevented the functioning of this enzyme. This time period corresponds to the healing period of TTN.

DLIS has a natriuretic effect in term and preterm neonates [18, 19, 38]. Our TTN group had higher serum Na^+^, osmolarity, diuresis, and FeNa^+^ levels, both on the 1st and 7th days, compared to the control group. The 1st day plasma DLIS was positively correlated with FeNa^+^ and diuresis and negatively correlated with serum osmolarity in TTN group but not in control infants. These findings may be explained as a compensatory natriuretic and diuretic effect of DLIS on the fluid and electrolyte balance in TTN.

Atrial natriuretic peptide (ANP) was found to be higher in RDS cases, correlating with the severity and progress of the disease and acting as an endogenous diuretic [39–41]. In contrast to these findings, $\ddot{\text{O}}$nal et al. [42], in a small group of TTN patients, showed that serum ANP concentrations were decreased compared to healthy infants. N-terminal pro-B-type natriuretic peptide (NT-proBNP), another member of natriuretic peptide family, was found higher in correlation with disease severity in TTN patients [14]. Increased DLIS levels, as shown in this study, can be important in the pathogenesis and course of TTN and may have a similar compensatory natriuretic effect.

Plasma DLIS levels were related with creatinine levels in adult [16], pediatric [43], and neonatal studies [44]. However, no correlation was found between the DLIS and creatinine levels of adult cases with renal insufficiency [45]. Our TTN group had similar urea and creatinine values with controls, and plasma DLIS levels were not correlated with renal functions of TTN patients. Thus, we may conclude that TTN and increased DLIS levels have no severe effect on the renal functions.

Important hemodynamic changes occur in early postnatal period [46, 47]. In a study of LV functions in about 20 healthy term infants, the 1st day LVEDD (3.18 ± 0.4 mL) and EF values (0.55 ± 0.05) were found to be statistically different than the 5th day values (2.73 ± 0.4 mL, 0.49 ± 0.07; *P* < 0.01 for both), but HR and LVESD values were similar. The changes in LVEDD values were suggested to be related to the PDA flow [47]. By studying RV functions in 18-term healthy newborns, RVEDD, RVESD, RVCO, and HR parameters were found to be similar on the 1st and 3rd days [48]. In our control group, only EF values showing LV functions changed, whereas, similar to previous study [48], no change was observed in RV functions during the study period.

Very few studies investigated the effect of TTN on cardiac functions. Clinically classifying TTN cases into classical (*n* = 19) and severe (*n* = 6) forms, classical cases were reported to have only a mild LV dysfunction on the first day, whereas severe cases had significant LV and RV dysfunctions [7]. This situation was speculated to be related to fetal persistent circulation, which could not be proven in blood gas analysis. Similarly, the more severe RV dysfunction was related to the more severe TTN findings and to the more severe and longer standing LV dysfunction in the first four hours of life in 42-term TTN patients. It was thus concluded that TTN caused both RV and LV dysfunctions and that initial right ventricle systolic time interval (RVSTI) values may predict the severity of TTN (RR: 17.5, *P* < 0.001) [49]. Both RV and LV functions were disturbed in our TTN group, compared to the control group, both on the 1st and 7th days of life, showing that the cardiac influence still goes on even in the recovery period.

Urine DLIS levels were found to be related to LV functions (HR, *P* < 0.05; LVEDD, *P* < 0.01; FS, *P* < 0.01) in 34 adults [50]. Moreover, in 401 adult patients of ICU, plasma DLIS levels were correlated with CO (*P* < 0.01), LV stroke volume (*P* < 0.01), and LV stroke strength (*P* < 0.01) [16]. In an experimental study, rats with cardiac straining were noted to have increased DLIS levels [51]. However, the relationship between plasma DLIS levels and cardiac functions has not been evaluated in newborns so far. In our study, plasma DLIS levels were significantly correlated with CO, LVEDD, and RVESD levels in the TTN group on the 1st day of life, showing the effect of DLIS on the initial phase of the disease. 

Na^+^-K^+^-ATPase and intracellular calcium have a negative effect on myocardial functions. Therefore, Na^+^-K^+^-ATPase inhibitor digoxin is effectively used in this treatment [52]. Increased DLIS levels may have a role in the pathophysiology of TTN, thus, negatively influencing the respiratory system-cardiac function relations or this increase may be a secondary compensation mechanism to restore the already disturbed cardiac functions in TTN.

In adults, mBP is related to DLIS [53, 54]. Initially, TTN cases had higher BP than controls, and none of the patients required treatment; BP values were more or less similar on the 7th day. This finding may be related to the acute effect of TTN on hemodynamic system. However, plasma DLIS levels were not correlated with mBP during any study period.

In the present study, for the first time in the literature, higher DLIS levels in correlation with serum osmolarity, diuresis, FeNa^+^ levels, and severity of disease have been shown in TTN cases. This correlation may indicate that it has a role in the pathophysiology of TTN, but more possibly DLIS levels may have increased as a compensatory mechanism for the volume overload caused due to the already disturbed LV and RV functions.

## 5. Conclusions

TTN patients have increased plasma DLIS levels in correlation with disease severity. Its cardiac effect may be a compensatory digoxin-like effect; besides, its natriuretic and diuretic effect may help in an already increased volume load status. However, this compensatory increase may cause a vicious cycle since it inhibits alveolar Na^+^-K^+^-ATPase and causes a further delay in the fetal lung fluid absorption.

## Figures and Tables

**Table 1:** Demographic and clinical characteristics of TTN and control groups.

	TTN (*n* = 15)	Control (*n* = 15)	*P*
Gestational age (week)	37.4 ± 0.9	36.9 ± 0.9	>0.05
(Mean ± SD) (min.–max.)	(36–39)	(35–38)	
Birthweight (gram)	2883.3 ± 182.4	2874.0 ± 264	>0.05
(Mean ± SD) (min.–max.)	(2550–3120)	(2450–3420)	
Delivery type (C/S) (*n*%)	10 (66.67%)	10 (66.67%)	>0.05
Sex (male) (*n*%)	9 (60%)	9 (60%)	>0.05
Apgar 1st (median) (min.–max.)	8 (7–10)	8 (7–10)	>0.05
Apgar 5th (median) (min.–max.)	9 (8–10)	9 (8–10)	>0.05
Arterial blood gas pH	7.32 ± 0.06	7.34 ± 0.04	>0.05
(Mean ± SD) (min.–max.)	(7.21–7.41)	(7.21–7.38)	
Antenatal fluid to mother (mL/kg/hour)	9.2 ± 6.8	13.0 ± 7.5	>0.05
(Mean ± SD) (min.–max.)	(0–21)	(0–26)	
Antenatal steroids (*n*%)	8 (53.33%)	7 (46.6%)	>0.05
CRP (mg/dL)	0.39 ± 0.23	0.44 ± 0.19	>0.05
(Mean ± SD) (min.–max.)	(0.10–0.80)	(0.21–0.70)	
Sepsis or pneumonia (*n*%)	0	0	>0.05
Time of inclusion (hour)	14.86 ± 2.23	16.13 ± 2.67	>0.05
	(12–20)	(12–22)	
RR_0 _(/min.ute)	72.2 ± 9.0	47.6 ± 2.9	<0.05
(Mean ± SD ) (min.–max.)	(60–90)	(44–54)	
RR_max._(/min.ute)	87.0 ± 9.6	52.0 ± 2.5	<0.05
(Mean ± SD) (min.–max.)	(65–105)	(48–56)	
Duration of tachypnea (DoT) (hour)	90.0 ± 23.4	—	—
(Mean ± SD) (min.–max.)	(48–144)		
Respiratory assistance (*n*%)		—	—
Only O_2 _support	11 (73.3%)		
nCPAP	2 (13.3%)		
Mechanical ventilation	2 (13.3%)		
Duration of hospitalization (hour)	148.2 ± 47.8	30.2 ± 11.4	<0.05
(Mean ± SD) (min.–max.)	(240–926)	(16–120)	
Pneumothorax (*n*%)	2 (13.3%)	—	—
DLIS_1 _(ng/mL)	0.66 ± 0.37	0.24 ± 0.20	<0.01
(Mean ± SD) (min.–max.)	(0–1.30)	(0–0.80)	
DLIS_7 _(ng/mL)	0.27 ± 0.21	0.11 ± 0.11	<0.05
(Mean ± SD) (min.–max.)	(0–0.76)	(0–0.45)	

S/C: caesarian section.

CRP: serum C reactive protein.

RR_0_: respiratory rate on admission.

RR_max._: max.imum respiratory rate on follow-up.

CPAP: nasal continuous positive airway pressure.

DLIS_1_: digitalis-like immunoreactive substance at the 1st day.

DLIS_7_: digitalis-like immunoreactive substance at the 7th day.

**Table 2:** Evaluation of fluid and ion balance parameter on the 1st and 7th days of TTN and control groups.

Parameter*	TTN (*n* = 15)		Control (*n* = 15)		*P*			
	1st day^a^	7th day^b^	1st day^c^	7th day^d^	a versus b	c versus d	a versus c	b versus d
Na^+^ (meq/L)	143.0 ± 4.1	139.7 ± 5.2	139.3 ± 5.1	135.6 ± 4.6	<0.05	<0.05	<0.05	<0.05
	(138–154)	(128–148)	(132–148)	(128–141)				
K^+^ (meq/L)	3.7 ± 0.4	3.7 ± 0.3	4.0 ± 0.2	3.9 ± 0.3	>0.05	>0.05	<0.05	>0.05
	(3.2–4.6)	(3.2–4.4)	(3.7–4.4)	(3.2–4.3)				
Urea (mg/dL)	28.7 ± 8.1	28.6 ± 7.8	30.4 ± 8.6	40.2 ± 9.9	>0.05	<0.05	>0.05	<0.01
	(20–42)	(18–43)	(18–43)	(30–65)				
Creatinine (mg/dL)	0.88 ± 0.20	0.80 ± 0.21	0.88 ± 0.17	0.85 ± 0.21	>0.05	>0.05	>0.05	>0.05
	(0.50–1.23)	(0.45–1.20)	(0.55–1.20)	(0.56–1.30)				
Blood osmolarity (mosm/L)	304.1 ± 6.9	297.3 ± 7.5	295.8 ± 10.1	294.7 ± 9.4	<0.05	>0.05	<0.05	>0.05
	(295–316)	(285–308)	(275–306)	(280–311)				
Urine osmolarity (mosm/L)	403.3 ± 43.5	364.0 ± 54.9	402.8 ± 43.8	402.7 ± 40.6	>0.05	>0.05	>0.05	<0.05
	(320–490)	(245–440)	(330–468)	(324–460)				
FeNa^+^ (%)	2.47 ± 0.26	1.79 ± 0.25	1.76 ± 0.34	1.51 ± 0.29	<0.001	<0.01	<0.001	<0.01
	(2.10–2.89)	(1.32–2.20)	(1.20–2.65)	(1.0–2.11)				
Diuresis (mL/kg/hour)	3.12 ± 0.82	2.05 ± 0.41	2.34 ± 0.82	2.19 ± 0.89	<0.001	>0.05	<0.05	>0.05
	(2.0–4.3)	(1.5–2.8)	(1.1–4.4)	(1.3–4.4)				

*Values are represented as mean ± SD (min.–max.).

**Table 3:** Evaluation of hemodynamic and echocardiogram parameter on the 1st and 7th days of TTN and control groups.

Parameter*	TTN (*n* = 15)		Control (*n* = 15)		*P*			
	1st day^a^	7th day^b^	1st day^c^	7th day^d^	a versus b	c versus d	a versus c	b versus d
CO (mL/minute)	332.0 ± 34.5 (256–398)	312.8 ± 25.8 (277–345)	302.4 ± 24.9 (245–345)	301.2 ± 22.3 (256–334)	<0.01	>0.05	<0.05	>0.05
EF (%)	51.1 ± 1.0 (48–55)	49.0 ± 1.0 (47–53)	49.0 ± 2.0 (47–55)	43.0 ± 3.0 (43–54)	<0.01	<0.05	<0.05	>0.05
LVEDD (mm/kg)	30.76 ± 1.63 (28.0–33.2)	29.44 ± 1.05 (27.6–31.2)	29.73 ± 1.18 (28.0–33.0)	29.38 ± 0.79 (28.4–31.8)	<0.01	>0.05	<0.05	>0.05
LVESD (mm/kg)	14.66 ± 1.32 (12.9–17.6)	12.92 ± 1.23 (10.7–14.5)	12.18 ± 0.82 (10.7–13.1)	11.89 ± 0.72 (10.5–12.8)	<0.001	>0.05	<0.001	<0.01
RVEDD (mm/kg)	24.26 ± 2.25 (21.2–27.5)	23.66 ± 2.72 (19.5–27.4)	21.34 ± 2.0 (18.8–24.5)	20.88 ± 1.47 (18.7–24.2)	<0.05	>0.05	<0.01	<0.01
RVESD (mm/kg)	11.13 ± 0.92 (10.2–13.0)	10.18 ± 0.84 (8.5–11.3)	10.34 ± 1.01 (8.0–12.1)	9.78 ± 1.26 (8.0–12.0)	<0.05	>0.05	<0.05	>0.05
LA/Aorta	1.43 ± 0.12 (1.21–1.68)	1.15 ± 0.08 (0.99–1.26)	1.12 ± 0.09 (1.0–1.30)	1.12 ± 0.08 (1.0–1.30)	<0.001	>0.05	<0.001	>0.05
FS (%)	30.26 ± 2.05 (27.0–34.0)	30.20 ± 2.23 (27.0–35.0)	31.0 ± 2.1 (28.0–35.0)	30.6 ± 1.8 (28.0–34.0)	>0.05	>0.05	>0.05	>0.05
HR (/minute)	159.6 ± 7.21 (148–172)	146.33 ± 8.55 (133–165)	144.0 ± 10.8 (118–156)	138.7 ± 12.0 (110–153)	<0.001	>0.05	<0.001	>0.05
mBP (mmHg)	52.7 ± 6.05 (44–65)	49.5 ± 6.6 (40–64)	46.3 ± 5.03 (40–57)	49.4 ± 5.5 (41–59)	>0.05	>0.05	<0.01	>0.05

*Values are represented as mean ± SD  (min.–max.).
